# Prospective Associations of Dietary Antioxidant Vitamin Intake and 8-Year Risk of Elevated Serum *C*-Reactive Protein Levels

**DOI:** 10.3390/nu17061020

**Published:** 2025-03-14

**Authors:** Inkyung Baik

**Affiliations:** Department of Foods and Nutrition, College of Science and Technology, Kookmin University, Seoul 02707, Republic of Korea; ibaik@kookmin.ac.kr; Tel.: +82-2-910-4774

**Keywords:** vitamin A, vitamin C, vitamin E, *C*-reactive protein, prospective studies

## Abstract

Background/Objectives: Circulating high-sensitivity *C*-reactive protein (hsCRP) is a well-established biomarker of low-grade systemic inflammation; levels above 3 mg/L indicate high cardiovascular risk. Although cross-sectional studies have shown associations between antioxidant vitamin intake and hsCRP levels, prospective data remain limited. This study aims to investigate the associations of dietary intake of vitamins A, C, and E with the 8-year risk of elevated serum hsCRP levels (>3 mg/L). Participants/Methods: This prospective study included 7695 adults from population-based cohorts. Serum hsCRP was assayed at the 4- and 8-year follow-ups; levels above 3 mg/L were considered elevated. Dietary intake of vitamin A, retinol, β-carotene, and vitamins C and E was assessed at baseline and at the 4-year follow-up using a food frequency questionnaire. A multivariable Cox proportional hazards regression was conducted with adjustments for potential confounders. Results: When vitamin intake was categorized into quintiles, vitamin C intake demonstrated an inverse association, whereas β-carotene intake exhibited a U-shaped association with the risk of elevated serum hsCRP concentrations. Hazard ratios (HRs) [95% confidence intervals (CIs)] for the third and fourth quintiles of vitamin C intake were 0.72 [0.53, 0.98] and 0.70 [0.49, 0.98], respectively, compared with the first quintile. The HR [95% CI] for the third quintile of β-carotene intake was 0.69 [0.50, 0.95] compared with the first quintile. However, excessive consumption of vitamin E increased the risk of elevated hsCRP levels; HR (95% CI) was 1.62 [1.19, 2.21] for participants consuming >120% of adequate intake (AI) relative to those with 80–119% of AI. In stepwise analysis to identify a best-fit model, significant variables included the presence of diabetes or hypertension, calorie intake, age, body mass index, sex, educational level, moderate or vigorous physical activity, and vitamin C intake. Conclusion: These findings suggest that dietary intake of vitamins A and C may help prevent elevated hsCRP levels in the general adult population. Further epidemiological studies are warranted to confirm these potential causal associations.

## 1. Introduction

*C*-reactive protein (CRP), a nonspecific systemic marker of inflammation, is synthesized in the liver in response to inflammatory stimuli. Its circulating concentration substantially increases to levels above 10 mg/L in response to acute and chronic conditions, including infection, inflammation, and tissue damage [1,2]. Given the widespread use of high-sensitivity CRP (hsCRP) assays, which detect lower CRP concentrations than standard assays, numerous epidemiological studies have shown that hsCRP serves as a valuable marker for predicting the risk of cardiovascular disease (CVD) [3]. Consequently, classifications of circulating hsCRP concentrations as <1 mg/L, 1–3 mg/L, and >3 mg/L, corresponding to low, moderate, and high cardiovascular risk, respectively, have been proposed for CVD risk assessments [4].

Multiple lifestyle factors, including diet, have been associated with low-grade inflammation as measured by CRP or hsCRP. Both cross-sectional and longitudinal studies have shown that smoking is associated with elevated hsCRP levels [5,6], whereas physical activity is linked to lower levels [6,7]. Body mass index (BMI) has demonstrated a cross-sectional association with higher hsCRP concentrations [8], and longitudinal changes in BMI have been positively correlated with changes in CRP concentrations [9]. Dietary factors, particularly dietary antioxidant nutrient intake and vegetable and fruit consumption, have been linked to hsCRP concentrations in cross-sectional studies [8,10,11] and in a longitudinal study [12]. A cross-sectional study based on a U.S. national survey revealed an inverse association between dietary intake of vitamins A, C, and E and elevated serum CRP levels (>3 mg/L) [10]. Regarding food consumption, fruit and vegetable consumption was associated with lower hsCRP levels [8,11]. Additional cross-sectional studies have examined associations with antioxidant biomarkers in the blood [13,14,15,16]. Adults with higher concentrations of serum vitamins A and C have shown lower CRP levels across various ethnic groups [13,14,15,16]. A single longitudinal study assessing changes in hsCRP concentrations over 3 years has identified significant associations between higher vegetable and blueberry intake and reduced hsCRP concentrations [12]. However, longitudinal data investigating temporal associations between antioxidant vitamin status and CRP or hsCRP concentrations have not yet been reported.

This prospective study aims to investigate the association between dietary intake of vitamins A, C, and E and the incidence of elevated serum hsCRP levels (>3 mg/L) over an 8-year period. Other risk factors associated with elevated hsCRP levels and their potential modifying effects on the relationship between antioxidant dietary intake and hsCRP concentrations are also explored.

## 2. Participants and Methods

### 2.1. Participants

The study population consisted of male and female adults enrolled as cohort members of the Korean Genome Epidemiology Study, administered by the Korea National Institute of Health (KNIH). Specifically, participants were exclusively drawn from two cohorts, referred to as the “Ansung cohort” and the “Ansan cohort”, both of which are ongoing population-based prospective studies. Detailed descriptions of the study population, design, methods, and procedures have been provided in previous reports [17,18]. Briefly, adult residents of Ansung and Ansan cities (Gyeonggi Province, Republic of Korea) were enrolled using a two-stage cluster sampling method. Participants were registered after completion of an initial onsite interview and a comprehensive health examination (conducted between June 2001 and January 2003). Subsequently, follow-up interviews and health examinations were performed through periodic site visits. At the initial and follow-up visits, participants provided informed consent through a process approved by the Institutional Review Board of the study site. The raw data utilized in this study were distributed by the KNIH National Biobank of Korea (https://biobank.nih.go.kr/eng, accessed on 13 February 2025). Institutional Review Board approval from Kookmin University (KMU-202312-HR-384) was also obtained to secure KNIH’s permission for data release.

Because dietary information was collected exclusively at baseline and during the follow-up period between April 2005 and November 2006, this period was designated as the first 4-year follow-up. Accordingly, the second follow-up was defined as the subsequent 4-year period between April 2009 and December 2010. Although follow-up data from 2011 to 2020, except for dietary data, are available, only data collected between 2001 and 2010 were used in this study. This selection was based on considerations related to loss to follow-up, an appropriate exposure–outcome period, and potential changes in dietary intake over an extended follow-up period.

Considering the baseline data for 10,030 cohort members, only those with serum CRP concentrations ≤ 3 mg/L (*n* = 8192) were included in the present study. Of these, 330 participants were excluded due to improper dietary data (missing data or total calorie intake < 500 kcal/day or >5000 kcal/day) or missing BMI data. To eliminate potential cases of acute infection, including bacterial and viral infections, an additional 167 participants with serum hsCRP levels exceeding 10 mg/L were excluded throughout the 8-year follow-up period. Ultimately, data from 7695 participants (3606 men and 4089 women) were analyzed.

### 2.2. Outcome Definition

The primary outcome was the serum hsCRP level, specifically cases with elevated levels exceeding 3 mg/L. All serum samples were collected in the morning after overnight fasting and transported immediately to a commercial laboratory for biochemical assays; quality control and commutability of assay data were ensured by KNIH. Standard CRP assays were conducted for baseline samples, whereas hsCRP assays were performed for follow-up samples using automated analyzers (CRP: Hitachi Automatic Analyzer 7600, Hitachi, Nittobo, Japan; hsCRP: ADVIA 1650, Siemens, Tarrytown, NY, USA).

### 2.3. Dietary Antioxidant Vitamin Consumption

Dietary information was collected using a food frequency questionnaire (FFQ) developed and validated by the KNIH [18]. The FFQ includes data regarding the average consumption frequency and serving size for each food item (103 items at baseline and 106 items at follow-up) to assess food intake over the previous year. Nine consumption frequency categories, ranging from “almost never” to “three or more times per day”, and three serving size options, smaller, equal to, or larger than a standard serving size, were listed. The average frequency of each food item was calculated by multiplying the reported consumption frequency by 0.5 for smaller portions, 1 for equal portions, or 1.5 for larger portions relative to the standard serving size. For seasonal fruits, additional inquiries regarding consumption frequency during specific periods were included and factored into the calculation. Average daily nutrient intake was calculated using FFQ responses and a food composition database published by the Rural Development Administration of Korea [19]. The KNIH provided dietary data for 23 nutrients, including total calories, carbohydrates, protein, fat, fiber, six minerals, and ten vitamins, such as vitamin A, retinol, β-carotene, and vitamins C and E. These antioxidant vitamins constituted the primary exposures in this study.

### 2.4. Potential Confounding Variables

Data regarding demographic and health-related factors, including age, sex, residential district, educational level, income level, occupation, BMI (calculated as body weight [kg] divided by height squared [m^2^]), smoking status, alcohol consumption, moderate or vigorous physical activity, the presence of diabetes mellitus (DM) or hypertension (HTN), history of CVD or cancer diagnosis, and use of dietary supplements (vitamins, minerals, and functional foods) were collected and considered potential confounding variables in the analysis.

### 2.5. Statistical Analysis

Descriptive statistics were calculated according to hsCRP quartile groups and are presented as the mean ± standard deviation or percentage. Statistical differences across groups were assessed using analysis of variance for continuous variables and the chi-square test for categorical variables. To evaluate associations between dietary antioxidant vitamin intake and the 8-year risk of elevated hsCRP concentrations (>3 mg/L), Cox proportional hazards regression and the Efron approximation method were utilized. Hazard ratios (HRs) and 95% confidence intervals (CIs) were estimated. Person-years were calculated from the date of each participant’s baseline examination to the last follow-up date. In multivariable models, age, BMI, and calorie intake were included as continuous variables; sex, residential district (urban or rural), educational level (≤middle school or ≥high school), income level (monthly income ≤ 2 × 10^6^ Won or >2 × 10^6^ Won), occupation (office work or other), smoking status (never smoked, ≤20 pack-years, or >20 pack-years), alcohol consumption (abstinent, <15 g/day, 15–30 g/day, or >30 g/day), moderate or vigorous physical activity (quintiles of metabolic equivalent (MET)-hours/day), dietary supplementation (yes or no), total calorie intake, the presence of DM or HTN (yes or no), and history of CVD or cancer diagnosis (yes or no) were included as categorical variables. Notably, data regarding BMI, smoking status, moderate or vigorous physical activity, the presence of DM or HTN, history of CVD or cancer diagnosis, and nutrient intake were updated using information from the first follow-up. Baseline information was used for variables that were less likely to change over time or that could not be updated due to insufficient data. Total calorie intake was adjusted in the model using the energy adjustment method, rather than the residual method [20], because actual dietary antioxidant vitamin consumption was needed for comparison with dietary reference intake (DRI) values [21]. In further analysis, the stepwise method was applied to select significant variables and identify a best-fit model with the lowest Akaike information criterion (AIC) value. Proportional hazards assumptions were tested in a full model that included all potential confounding factors, and no violations were detected. Additionally, multivariable association analyses stratified by significant variables selected through the stepwise method were conducted. All statistical analyses were performed using the SAS software (SAS 9.4, SAS Institute, Cary, NC, USA) with a two-sided significance (*p* < 0.05).

## 3. Results

### 3.1. Study Participants Characteristics

Among the 7695 study participants, 649 (8.4%) had elevated serum CRP concentrations (>3 mg/L). Table 1 presents a comparison of study participants according to quartile groups of baseline CRP concentrations. Participants in the highest quartile group were more likely to be older (*p* for trend < 0.001), reside in rural areas (*p* for trend < 0.05), have a lower educational attainment (*p* for trend < 0.05) and income level (*p* for trend < 0.01), have higher BMI (*p* for trend < 0.001), smoke heavily (*p* for trend < 0.05), have DM or HTN (*p* for trend < 0.001), and consume fewer calories (*p* for trend < 0.05) and lower amounts of vitamins A (*p* for trend < 0.05), C (*p* for trend < 0.01), and E (*p* for trend < 0.05).

### 3.2. Associations Between Dietary Antioxidant Vitamin Intake and the Risk of Elevated hsCRP Concentrations (>3 mg/L)

Table 2 presents the associations between dietary intake of vitamins A, C, and E and the 8-year risk of elevated hsCRP concentrations (>3 mg/L). In the multivariable model 2, potential confounding variables from model 1 were adjusted for, along with antioxidant vitamin intake. In model 1, participants in the third quintile groups for vitamin A (range: 380–502 RAE/day), retinol (range: 44–67 μg/day), and β-carotene (range: 1867–2481 μg/day) intake exhibited a significantly lower risk of elevated hsCRP concentrations compared with their respective reference groups. In model 2, after further adjustment for vitamin C and E intake, the significant associations for vitamin A and β-carotene intake persisted. For vitamin C and E intake, participants in the second, third, and fourth quintile groups exhibited a significantly lower risk in model 1. In model 2, inverse associations remained significant for the third (range: 89–116 mg/day) and fourth (range: 116–158 mg/day) quintile groups of vitamin C intake. These findings suggest that dietary intake of vitamins A and C reduces the risk of elevated hsCRP concentrations (>3 mg/L), independently of other antioxidant vitamins. The association for vitamin C followed a linear trend, whereas vitamin E exhibited a nonlinear U-shaped association.

### 3.3. Associations Between DRI-Based Antioxidant Vitamin Intake and the Risk of Elevated hsCRP Concentrations (>3 mg/L)

**Figure 1:**
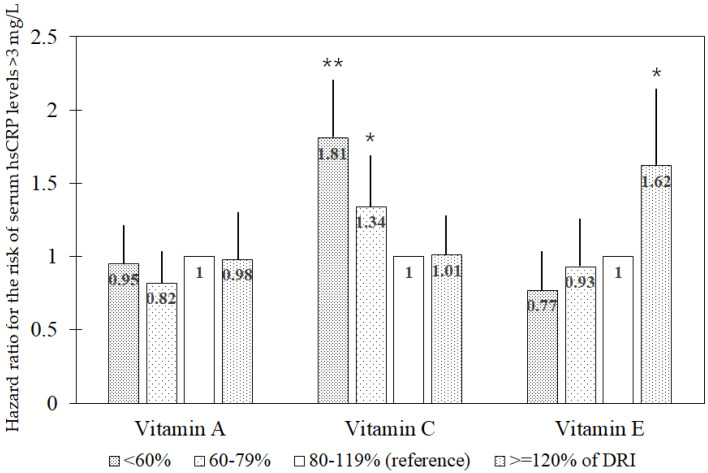
Multivariable hazard ratio for the risk of elevated serum high-sensitivity *C*-reactive protein concentrations (>3 mg/L) in the associations of DRI^1^-based groups (<60%, 60–79%, 80–119%, and ≥120% of DRI) for antioxidant vitamins. Abbreviations: DRI, dietary reference intake; hsCRP, high-sensitivity *C*-reactive protein. The multivariable model includes age, sex, residential district (urban or rural), educational level (≤middle school or ≥high school), income level (monthly income ≤ 2 × 10^6^ Won or >2 × 10^6^ Won), occupation type (office work or other), body mass index, pack-years of cigarettes (0, <20, or ≥20 pack-years), alcohol consumption (abstinent, <15 g/day, 15–30 g/day, or >30 g/day), moderate or vigorous physical activity (quintiles of metabolic equivalent-hours/day), dietary supplementation (yes or no), total calorie intake, the presence of diabetes mellitus or hypertension (yes or no), and history of cardiovascular disease or cancer (yes or no), along with dietary intake of vitamins A, C, and E. ^1^ Recommended daily allowance for vitamins A and C and adequate intake for vitamin E. * *p* < 0.05; ** *p* < 0.001.

As shown in Figure 1, the associations between DRI-based antioxidant vitamin intake and the 8-year risk of elevated hsCRP concentrations (>3 mg/L) are illustrated. The recommended daily allowance (RDA) for vitamin A is 800 retinol activity equivalents (RAE) for men aged 30–49 years, 750 RAE for men aged 50–64 years, 700 RAE for men aged ≥65 years, 650 RAE for women aged 30–49 years, and 600 RAE for women aged ≥50 years. The RDA for vitamin C is 100 mg for adults aged ≥30 years and the DRI for vitamin E is 12 mg α-tocopherol equivalent, defined as adequate intake (AI). Based on these DRI values, intake was categorized into four groups: <60%, 60–79%, 80–119%, and ≥120% of the DRI. The risk of elevated hsCRP concentrations was analyzed across these groups. Participants with vitamin C intake below the recommended level exhibited significantly higher risks than those consuming 80–119% of the RDA. HRs (95% CIs) were 1.81 (1.38, 2.38) and 1.34 (1.03, 1.74) for <60% and 60–79% intake groups, respectively, compared with the 80–119% group. However, no significant association was observed for vitamin A intake. In contrast, vitamin E intake exceeding 120% of AI significantly increased the risk of elevated hsCRP concentrations with an HR (95% CI) of 1.62 (1.19, 2.21) for the >120% intake group relative to the 80–119% group.

### 3.4. Stepwise Analysis of Associations Between Risk-Related Variables and the Risk of Elevated hsCRP Concentrations (>3 mg/L)

Table 3 presents the association results for significant risk-related variables in relation to the risk of elevated hsCRP concentrations (>3 mg/L) identified through the stepwise selection method. Variables were ranked based on statistical significance; the presence of DM or HTN, calorie intake, age, BMI, sex, educational level, moderate or vigorous physical activity, and vitamin C intake were selected. This model yielded the lowest AIC value (10,871.29), indicating a best-fit model. Among modifiable factors, moderate or vigorous physical activity, vitamin C intake, lower calorie intake, and lower BMI were associated with a significantly reduced risk.

Table 4 presents the association results stratified by significant variables, including the presence of DM or HTN, age, BMI, sex, educational level, and physical activity, as identified in Table 3. The inverse association between vitamin C intake and the risk of elevated hsCRP concentrations was particularly prominent in men and in older, leaner, less educated, and less physically active individuals. Within these groups, a risk reduction exceeding 30% was observed. Among individuals without DM or HTN, those with higher BMI, and those with a higher educational attainment, only the second tertile group demonstrated a significant reduction in risk. In contrast, among individuals with DM or HTN, a significant risk reduction was observed exclusively in the third tertile group with higher vitamin C intake.

## 4. Discussion

This population-based prospective study examined associations between dietary antioxidant vitamin intake and the 8-year risk of elevated serum hsCRP concentrations (>3 mg/L). Overall, dietary vitamin C intake demonstrated an inverse association with the risk, whereas vitamin A intake, especially β-carotene intake, exhibited a U-shaped relationship with the lowest risk observed at moderate consumption levels. Based on the RDA, deficient vitamin C intake was associated with an approximately 30% to 80% increase in the risk compared with an intake of 80–119% of the RDA, independently of vitamins A and E intake. Furthermore, vitamin C intake was exclusively selected among antioxidant vitamins in the best-fit model for predicting risk, along with other significant risk factors, including the presence of DM or HTN, calorie intake, age, BMI, sex, educational level, and physical activity.

CRP is an acute-phase protein synthesized by hepatocytes in response to inflammatory cytokines, primarily interleukin-6 (IL-6), which has been identified as a regulator of CRP transcription [2]. Both CRP and IL-6 levels are positively correlated with markers of oxidative stress, which occurs when reactive oxygen species (ROS) production exceeds the body’s antioxidant capacity [22]. Conversely, meta-analyses of earlier clinical trials have shown that vitamin C supplementation reduces CRP levels, whereas vitamin A supplementation does not [23,24]. However, available data remain insufficient to establish causal inferences regarding the relationship between dietary antioxidant intake and CRP levels.

Several cross-sectional studies have examined associations between antioxidant vitamin status and CRP levels [10,13,14,15,16,25,26]. Data from different cycles of the U.S. national survey demonstrate an inverse association between dietary intake of vitamins A, C, and E and elevated CRP concentrations (>3 mg/L) [10], with vitamin intake levels decreasing as CRP concentrations increase [25]. Additionally, blood concentrations of vitamins A and C are inversely associated with CRP levels [13,14,15,16]; individuals with deficient or inadequate plasma vitamin C levels exhibit significantly higher CRP concentrations than those with higher normal levels [26]. Notably, one study has revealed that individuals with elevated hsCRP concentrations (3–10 mg/L) have significantly higher serum vitamin E levels than those with hsCRP concentrations < 0.5 mg/L [16].

The present prospective study conducted a novel investigation to identify significant risk factors associated with elevated hsCRP concentrations (>3 mg/L) from a broad range of demographic and lifestyle variables. Based on stepwise analysis results, the most influential risk factor was the presence of DM and HTN. Previous studies have already demonstrated a strong association between CRP levels and the development of DM [27] and HTN [28]. The findings of this study are consistent with previous data regarding age and BMI but do not align with earlier reports on sex differences [8]. A cross-sectional study indicates that women are more likely to have higher CRP levels [8], whereas the present study identified the male sex as a risk factor for elevated CRP concentrations; this finding matches previous data of sex differences in CVD incidence [29]. Additionally, considering that 92% of current smokers in this study were men, smoking behavior may have contributed to the observed association between the male sex and elevated CRP concentrations; smoking is more likely to contribute to CVD risk in men [29]. Findings related to educational level and physical activity are consistent with previous reports [7,30]. In further analyses stratified by these risk factors, the inverse associations between vitamin C intake and the risk of elevated CRP concentrations were particularly prominent among individuals with higher risk profiles, including older adults, men, individuals with a lower educational attainment, and those with lower physical activity levels. Furthermore, among individuals without DM or HTN, a significant risk reduction was observed exclusively in the second tertile group of vitamin C intake; among individuals with DM or HTN, a significant risk reduction was observed exclusively in the third tertile group with higher vitamin C intake.

With respect to the DRI-based findings of the present study regarding vitamin E, consumption exceeding 120% of AI was associated with an increased risk of elevated hsCRP concentrations relative to intake in the 80–119% range in the model adjusted for vitamins A and C intake. Biological mechanisms underlying this association are unclear; because soybean oil is a major dietary source of vitamin E in Korea [31], high vitamin E intake may reflect high consumption of soybean oil, of which polyunsaturated fatty acids are highly susceptible to peroxidation during oil heating [32] reducing vitamin E content [32].

The strengths of this study include its prospective design, analysis of data from population-based cohorts, and consideration of a wide range of potential confounding variables. However, some limitations should be acknowledged when interpreting these findings. First, because dietary information was collected using an FFQ, absolute nutrient intake could not be determined; measurement errors related to the limited food list and frequency and portion size options cannot be excluded. Second, selection bias due to loss to follow-up (32%) may have occurred, although baseline antioxidant vitamin intake did not differ between participants who completed the follow-up and those who were lost. Third, residual confounding due to unmeasured variables is possible. Finally, the generalizability of these findings is limited.

## 5. Conclusions

This study identified an inverse association between dietary vitamin C intake and the 8-year risk of elevated hsCRP concentrations (>3 mg/L), even after adjustments for vitamins A and E intake and other risk factors. This association was more pronounced among individuals with established risk factors. Dietary intake of vitamin A, particularly β-carotene exhibited a nonlinear U-shaped relationship; the lowest risk was observed at moderate intake levels. In the DRI-based analysis, excessive vitamin E intake was associated with an increased risk relative to adequate intake, suggesting that caution is necessary regarding high consumption. Further epidemiological studies are needed to confirm these findings. Efforts to ensure sufficient vitamin C intake remain important public health considerations for adults susceptible to inflammation.

## Figures and Tables

**Table 1:** Baseline characteristics of 7695 study participants according to quartile groups of serum *C*-reactive protein concentrations.

Variables		All	Serum CRP Quartiles (Q) [Median, mg/L]				*p* Value for Trend
			1st Q [0.1]	2nd Q [0.9]	3rd Q [1.4]	4th Q [2.2]	
Age, years		51.7 ± 8.8	51.0 ± 8.8	51.2 ± 8.7	52.0 ± 8.7	52.6 ± 8.9	<0.001
Men, %		46.9	45.9	46.0	46.8	48.6	0.074
Residential district:	urban, %	52.1	52.6	54.8	51.9	49.5	<0.05
	rural, %	47.9	47.4	45.2	48.1	50.5	
Educational level ≥ high school, %		44.6	46.0	45.7	44.3	42.5	<0.05
Low-income level ^1^, %		62.9	61.4	60.7	64.0	65.3	<0.01
Office worker, %		8.3	8.4	8.9	9.0	7.0	0.122
Body mass index, kg/m^2^		24.4 ± 3.0	23.8 ± 2.9	24.2 ± 3.0	24.4 ± 2.9	25.1 ± 3.1	<0.001
Current smoker, %		24.7	23.3	24.6	25.3	25.7	0.064
Pack-years of cigarettes		9.2 ± 15.6	8.8 ± 16.1	8.7 ± 14.9	9.4 ± 15.3	9.9 ± 15.8	<0.05
Current alcohol drinker, %		47.9	47.2	47.2	48.2	49.0	0.191
Physical activity ^2^, MET-hours/day		16.4 ± 17.6	16.3 ± 17.4	15.9 ± 17.5	16.6 ± 17.9	16.5 ± 17.6	0.511
Presence of DM or HTN, %		48.8	43.1	44.8	50.9	56.0	<0.001
History of CVD or cancer diagnosis, %		9.5	9.1	9.4	9.1	10.4	0.215
Dietary supplementation, %		19.0	20.1	17.8	19.4	18.8	0.561
Average daily dietary intake							
Calories, kcal		1881.8 ± 523.3	1897.9 ± 547.8	1892.4 ± 512.8	1872.7 ± 525.2	1864.6 ± 505.9	<0.05
Vitamin A, RAE		534.0 ± 389.4	555.5 ± 408.3	521.6 ± 377.7	535.4 ± 394.8	522.7 ± 375.0	<0.05
Retinol, μg		68.0 ± 61.8	70.7 ± 66.1	68.8 ± 63.6	68.9 ± 60.7	63.9 ± 56.4	<0.01
β-carotene, mg		2.7 ± 2.3	2.8 ± 2.4	2.7 ± 2.3	2.7 ± 2.3	2.7 ± 2.2	0.114
Vitamin C, mg		127.6 ± 93.7	134.5 ± 99.6	126.0 ± 97.0	124.7 ± 87.7	125.0 ± 89.6	<0.01
Vitamin E, mg		9.3 ± 4.9	9.6 ± 5.3	9.3 ± 4.6	9.3 ± 4.8	9.2 ± 4.6	<0.05

Abbreviations: CRP, *C*-reactive protein; MET, metabolic equivalent; DM, diabetes mellitus; HTN, hypertension; CVD, cardiovascular disease; RAE, retinol activity equivalent. Values are presented as the mean ± standard deviation or percentage. ^1^ Participants with a monthly income ≤2 × 10^6^ Won. ^2^ Moderate or vigorous physical activity.

**Table 2:** Associations between dietary antioxidant vitamin intake and the 8-year risk of elevated high-sensitivity *C*-reactive protein concentrations (>3 mg/L).

Antioxidant Vitamin Intake	Quintiles (Q)	Cases	Hazard Ratio (95% Confidence Interval)		
	[Median]	PY	Age-Adjusted	Multivariable Model 1	Multivariable Model 2
Vitamin A, RAE	1st Q [214]	142/10,212	Reference	Reference	Reference
	2nd Q [329]	120/10,909	0.82 (0.64, 1.04)	0.77 (0.60, 0.98)	0.92 (0.70, 1.21)
	3rd Q [438]	99/10,921	0.66 (0.51, 0.86)	0.59 (0.45, 0.77)	0.72 (0.52, 0.99)
	4th Q [580]	136/10,697	0.95 (0.75, 1.20)	0.79 (0.61, 1.02)	0.96 (0.68, 1.34)
	5th Q [890]	152/10,432	1.09 (0.87, 1.37)	0.84 (0.64, 1.11)	0.93 (0.64, 1.37)
Retinol, μg	1st [14]	145/10,287	Reference	Reference	Reference
	2nd [34]	122/10,796	0.86 (0.68, 1.10)	0.81 (0.63, 1.03)	0.87 (0.68, 1.12)
	3rd [55]	107/10,858	0.76 (0.59, 0.98)	0.72 (0.55, 0.93)	0.78 (0.60, 1.03)
	4th [79]	130/10,748	0.97 (0.76, 1.24)	0.86 (0.66, 1.11)	0.92 (0.70, 1.21)
	5th [124]	145/10,483	1.09 (0.86, 1.39)	0.92 (0.70, 1.22)	0.92 (0.69, 1.24)
β-carotene, μg	1st [1015]	141/10,251	Reference	Reference	Reference
	2nd [1610]	125/10,832	0.87 (0.68, 1.10)	0.80 (0.63, 1.02)	0.94 (0.72, 1.23)
	3rd [2139]	96/10,944	0.64 (0.50, 0.84)	0.57 (0.43, 0.74)	0.69 (0.50, 0.95)
	4th [2914]	139/10,665	0.98 (0.77, 1.24)	0.82 (0.63, 1.05)	0.97 (0.70, 1.33)
	5th [4721]	148/10,481	1.05 (0.83, 1.32)	0.82 (0.63, 1.07)	0.89 (0.62, 1.28)
Vitamin C, mg	1st [52]	146/10,174	Reference	Reference	Reference
	2nd [78]	120/10,731	0.77 (0.60, 0.98)	0.73 (0.57, 0.94)	0.83 (0.63, 1.09)
	3rd [101]	110/10,937	0.70 (0.55, 0.90)	0.64 (0.50, 0.83)	0.72 (0.53, 0.98)
	4th [133]	120/10,902	0.76 (0.60, 0.97)	0.68 (0.53, 0.88)	0.70 (0.49, 0.98)
	5th [202]	153/10,428	1.01 (0.81, 1.27)	0.83 (0.63, 1.09)	0.74 (0.51, 1.09)
Vitamin E, mg	1st [4.6]	147/10,360	Reference	Reference	Reference
	2nd [6.5]	101/10,798	0.69 (0.53, 0.89)	0.65 (0.50, 0.85)	0.78 (0.58, 1.05)
	3rd [8.1]	115/10,956	0.80 (0.62, 1.02)	0.71 (0.54, 0.92)	0.95 (0.68, 1.32)
	4th [10.1]	119/10,821	0.85 (0.66, 1.09)	0.73 (0.55, 0.98)	1.00 (0.68, 1.46)
	5th [14.1]	167/10,238	1.26 (1.01, 1.59)	1.00 (0.72, 1.40)	1.31 (0.83, 2.08)

Abbreviations: PY, person-years; RAE, retinol activity equivalent. Multivariable model 1 includes age, sex, residential district (urban or rural), educational level (≤middle school or ≥high school), income level (monthly income ≤ 2 × 10^6^ Won or >2 × 10^6^ Won), occupation type (office work or other), body mass index, pack-years of cigarettes (0, <20, or ≥20 pack-years), alcohol consumption (abstinent, <15 g/day, 15–30 g/day, or >30 g/day), moderate or vigorous physical activity (quintiles of metabolic equivalent-hours/day), dietary supplementation (yes or no), total calorie intake, the presence of diabetes mellitus or hypertension (yes or no), and history of cardiovascular disease or cancer (yes or no). Multivariable model 2 includes all variables from model 1, with additional adjustments for dietary intake of vitamins A, C, and E.

**Table 3:** Stepwise analysis of multivariable associations between risk-related variables and the 8-year risk of elevated high-sensitivity *C*-reactive protein concentrations (>3 mg/L).

Stepwise Selection	Selected Variables ^1^	Unit for	Reference Group for	Multivariable HR ^1^
		Continuous Variables	Categorical Variables	(95% CI)
1	Presence of DM or HTN		Absence	1.48 (1.25, 1.75)
2	Calorie intake	500 kcal		1.17 (1.07, 1.27)
3	Age	1 year		1.02 (1.01, 1.03)
4	Body mass index	1 kg/m^2^		1.06 (1.03, 1.09)
5	Men		Women	1.44 (1.22, 1.71)
6	Education ≤ middle school		≥High school	1.29 (1.07, 1.55)
7	Physical activity	5 MET-hours/day		0.97 (0.95, 0.99)
8	Vitamin C intake			
	2nd quintile		1st quintile	0.72 (0.56, 0.92)
	3rd quintile		1st quintile	0.64 (0.49, 0.82)
	4th quintile		1st quintile	0.65 (0.51, 0.85)
	5th quintile		1st quintile	0.80 (0.61, 1.06)

Abbreviations: HR, hazard ratio; CI, confidence interval; DM, diabetes mellitus; HTN, hypertension; MET, metabolic equivalent. The multivariable model for the stepwise procedure includes age, sex, residential district (urban or rural), educational level (≤middle school or ≥high school), income level (monthly income ≤ 2 × 10^6^ Won or >2 × 10^6^ Won), occupation type (office work or other), body mass index, pack-years of cigarettes (0, <20, or ≥20 pack-years), alcohol consumption (abstinent, <15 g/day, 15–30 g/day, or >30 g/day), moderate or vigorous physical activity (quintiles of metabolic equivalent-hours/day), dietary supplementation (yes or no), total calorie intake, the presence of diabetes mellitus or hypertension (yes or no), and history of cardiovascular disease or cancer (yes or no), along with dietary intake of vitamins A, C, and E. ^1^ Only selected variables are listed.

**Table 4:** Stratification analysis results for multivariable associations between vitamin C consumption and the 8-year risk of elevated high-sensitivity *C*-reactive protein concentrations (>3 mg/L).

Stratified Analysis	Groups	Multivariable HR (95% CI) for Vitamin C Tertiles		
		1st Tertile	2nd Tertile	3rd Tertile
Disease-stratified	Absence of DM or HTN	Reference	0.66 (0.44, 0.98)	0.91 (0.56, 1.46)
	Presence of DM or HTN	Reference	0.78 (0.59, 1.03)	0.62 (0.44, 0.89)
Age-stratified	<50 years	Reference	0.83 (0.57, 1.21)	0.99 (0.63, 1.56)
	≥50 years	Reference	0.65 (0.48, 0.87)	0.64 (0.45, 0.93)
BMI-stratified	<23 kg/m^2^	Reference	0.48 (0.30, 0.76)	0.41 (0.24, 0.72)
	≥23 kg/m^2^	Reference	0.76 (0.58, 0.99)	0.84 (0.60, 1.16)
Sex-stratified	Women	Reference	0.85 (0.60, 1.18)	1.03 (0.67, 1.58)
	Men	Reference	0.62 (0.45, 0.85)	0.63 (0.43, 0.93)
Education-stratified	≥High school	Reference	0.65 (0.45, 0.95)	0.84 (0.53, 1.32)
	≤Middle school	Reference	0.64 (0.48, 0.87)	0.66 (0.46, 0.96)
PA-stratified	≥Median value MET-hours/day	Reference	0.94 (0.68, 1.31)	0.95 (0.62, 1.45)
	<Median value MET-hours/day	Reference	0.56 (0.40, 0.77)	0.58 (0.39, 0.86)

Abbreviations: HR, hazard ratio; CI, confidence interval; DM, diabetes mellitus; HTN, hypertension; BMI, body mass index; PA, physical activity; MET, metabolic equivalent. The multivariable model includes age, sex, residential district (urban or rural), educational level (≤middle school or ≥high school), income level (monthly income ≤ 2 × 10^6^ Won or >2 × 10^6^ Won), occupation type (office work or other), body mass index, pack-years of cigarettes (0, <20, or ≥20 pack-years), alcohol consumption (abstinent, <15 g/day, 15–30 g/day, or >30 g/day), moderate or vigorous physical activity (quintiles of metabolic equivalent-hours/day), dietary supplementation (yes or no), total calorie intake, the presence of diabetes mellitus or hypertension (yes or no), and history of cardiovascular disease or cancer (yes or no), along with dietary intake of vitamins A, C, and E.

## Data Availability

Raw data are available through the National Biobank of Korea, following the data distribution procedure described on its website (https://biobank.nih.go.kr/eng, accessed on 13 February 2025).
